# Discrepancy Between Eligibility and Practice: A 14-Year Nationwide Study on Curative Resection in Elderly Hepatocellular Carcinoma Patients

**DOI:** 10.3390/cancers18152457

**Published:** 2026-07-30

**Authors:** Sun Woong Kim, Jun Sik Yoon, Young Lee, Heejoon Jang

**Affiliations:** 1 Division of Gastroenterology and Hepatology, Department of Internal Medicine, Veterans Health Service Medical Center, Seoul 05368, Republic of Korea; 2 Division of Gastroenterology and Hepatology, Department of Internal Medicine, Inje University Busan Paik Hospital, Inje University College of Medicine, Busan 47392, Republic of Korea; 3 VHS Medical Research Institute, Veterans Health Service Medical Center, Seoul 05368, Republic of Korea; 4 Department of Internal Medicine, Seoul National University College of Medicine, Seoul 03080, Republic of Korea; 5 Division of Gastroenterology and Hepatology, Department of Internal Medicine, Seoul Metropolitan Government Boramae Medical Center, Seoul 07061, Republic of Korea

**Keywords:** hepatocellular carcinoma, aged, hepatectomy, treatment disparities, Barcelona Clinic Liver Cancer, registries, survival analysis

## Abstract

Background/Objectives: The 2026 Barcelona Clinic Liver Cancer (BCLC) update recommends surgical resection for a single hepatocellular carcinoma larger than 2 cm with preserved liver function and states that age alone should not preclude surgery. How often eligible older patients undergo resection, and how they fare, remains unquantified. Methods: Using the Korean Primary Liver Cancer Registry (2008–2021), we identified 3374 patients meeting all registry-based eligibility criteria of the 2026 BCLC update. Survival was measured from the date of radiological diagnosis in every patient, with vital status and cause of death obtained by linkage to national death records. We compared use of resection and survival among patients younger than 75 years and those 75 years or older, and across resection, ablation, other treatment, and no treatment, using Cox regression, restricted mean survival time (RMST), and competing-risk models. Results: Resection was performed in 60.6% of patients younger than 75 years versus 28.9% of those 75 years or older (*p* < 0.001); among older patients not undergoing resection, 21.5% received none. Older age was independently associated with lower odds of resection (adjusted odds ratio 0.36, 95% CI 0.28–0.46; *p* < 0.001). In this age group, 5-year survival was 61.3%, 62.1%, 32.9%, and 6.5% across the four treatment categories; the adjusted hazard ratio for no treatment versus resection was 4.97 (95% CI 3.26–7.57; *p* < 0.001). In unadjusted analysis, mean survival within the first 5 years was 13.3 months longer after resection (RMST difference, 95% CI 9.7–16.9; *p* < 0.001). Findings were consistent in competing-risk, weighted, cirrhosis-adjusted, and multiply imputed analyses. Conclusions: Older patients meeting eligibility criteria underwent resection far less often than younger patients, and a substantial minority received none, a pattern associated with markedly shorter survival.

## Simple Summary

Liver cancer is increasingly diagnosed in older people. Guidelines recommend an operation to remove a single liver tumor larger than 2 cm when the liver still works well, and they state that age by itself should not rule out surgery. Using a nationwide Korean cancer registry, we studied patients who all met the same registry-based criteria for surgery and asked how often older patients actually had an operation and how they fared afterwards. Patients aged 75 years or older had surgery far less often than younger patients, and one in five of those who did not have surgery received no cancer treatment at all. Survival was longest after surgery and shortest among untreated patients, and the difference remained after accounting for competing causes of death. These findings suggest that treatment decisions in older adults should rest on liver function and overall health rather than on age alone.

## 1. Introduction

Hepatocellular carcinoma (HCC) is the third leading cause of cancer death worldwide [1]. In several Asian countries, hepatitis B vaccination and effective antiviral therapy have begun to reduce age-standardized incidence, yet the number of older patients with newly diagnosed HCC continues to rise. Korea illustrates this divergence: overall incidence has declined since 2008, but the number of patients 80 years or older is projected to quadruple between 2008 and 2028, reaching 21.3% of all HCC cases [2].

For patients who reach a curative stage, the Barcelona Clinic Liver Cancer (BCLC) system recommends surgical resection as first-line therapy for a single tumor larger than 2 cm with preserved liver function, normal bilirubin, no clinically significant portal hypertension (CSPH), and an Eastern Cooperative Oncology Group performance status (ECOG PS) of 0 in its 2026 update [3]; the Korean Liver Cancer Association-National Cancer Center (KLCA-NCC) guideline endorses the same recommendation [4]. Age is not among the criteria, and the available evidence indicates that age by itself does not limit the benefit of treatment. In a meta-analysis of resection for HCC, 5-year overall survival was 53.8% in elderly and 51.6% in younger patients [5], and among octogenarians it was 39.7%, with frailty rather than chronological age identifying those at risk [6]. In a Japanese nationwide survey of 12,587 hepatectomies, overall survival was lower in patients 75 years or older, yet recurrence-free survival and the cumulative incidence of liver-related deaths were almost identical across age groups, with the difference being driven by death from other causes [7]. The same pattern extends to systemic therapy, where atezolizumab plus bevacizumab is comparably effective and well tolerated in older adults, with poor outcomes largely confined to those with severely impaired hepatic reserve [8,9].

In practice, however, older patients are treated less aggressively. A meta-analysis of 103 studies and 154,152 patients found that older patients were less likely to receive curative treatment even when the disease was at an early stage, and that the difference between age groups widened rather than narrowed over the study periods examined [10]. Underuse of treatment in older patients has also been documented in the United States [11]. In a two-system US cohort, worse survival among older patients was associated with chronological age rather than with measured comorbidity burden [12], a finding that may reflect either age-related biology or the downstream effect of less aggressive treatment. The central difficulty is that most studies have not restricted their analyses to patients who clearly met every criterion for resection, leaving unresolved whether the difference reflects appropriate selection for comorbidity and frailty or a discrepancy between eligibility and practice.

We therefore used the Korean Primary Liver Cancer Registry (KPLCR) to examine the use of surgical resection among patients who met all registry-based eligibility criteria for resection under the BCLC system. We assessed how use of resection differed by age, identified factors independently associated with not undergoing resection, and compared overall survival across resection, ablation or liver transplantation, other treatment, and no treatment. We gave particular attention to older patients who received no treatment, a group rarely characterized despite earlier descriptions of untreated HCC in older Korean patients [13].

## 2. Materials and Methods

### 2.1. Study Design and Data Source

This retrospective cohort study followed the STROBE statement and its RECORD extension for studies using routinely collected health data [14]. The KPLCR is a nationwide cohort operated by the Korean Liver Cancer Association with the Korea Central Cancer Registry (KCCR), which collects clinical, tumor, and treatment data from a systematic random sample of newly diagnosed HCC cases nationwide and updates vital status annually through linkage to government death records [4,15]. We included consecutive adults 18 years or older registered between January 2008 and December 2021. Vital status and cause of death were complete through 31 December 2024.

### 2.2. Eligibility Criteria

We applied the surgical-eligibility criteria of the 2026 BCLC update [3]: a single intrahepatic HCC larger than 2 cm; no macrovascular or bile-duct invasion; no extrahepatic spread; no ascites; Child–Pugh class A; platelet count of at least 100 × 10^3^/μL; total bilirubin of 1.5 mg/dL or lower; and ECOG PS of 0, unknown, or missing, whereas patients with a documented PS of 1 or higher were excluded. Patients in whom ascites, platelet count, or bilirubin was not documented were excluded, so that eligibility was established only from recorded values. The guideline specifies preserved liver function without stating a numerical bilirubin threshold, so we set this criterion at 1.5 mg/dL or lower, a value that lies within Child–Pugh class A.

Hepatic venous pressure gradient is not recorded in the registry. We therefore used the combination of absent ascites, Child–Pugh class A, and a platelet count of at least 100 × 10^3^/μL as a surrogate for the absence of CSPH. Measurement of the hepatic venous pressure gradient is the reference standard, and current guidance recommends non-invasive assessment based on platelet count and liver stiffness when direct measurement is not available [16]. The registry records neither pressure measurements nor liver stiffness, so our definition is an approximation that cannot be validated internally; its limitations are addressed in the Discussion. The KLCA-NCC guideline similarly lists a platelet count below 100 × 10^3^/μL together with splenomegaly, varices, or ascites as indicators of portal hypertension, and regards thrombocytopenia as the most clinically relevant of these [4].

**Figure 1:**
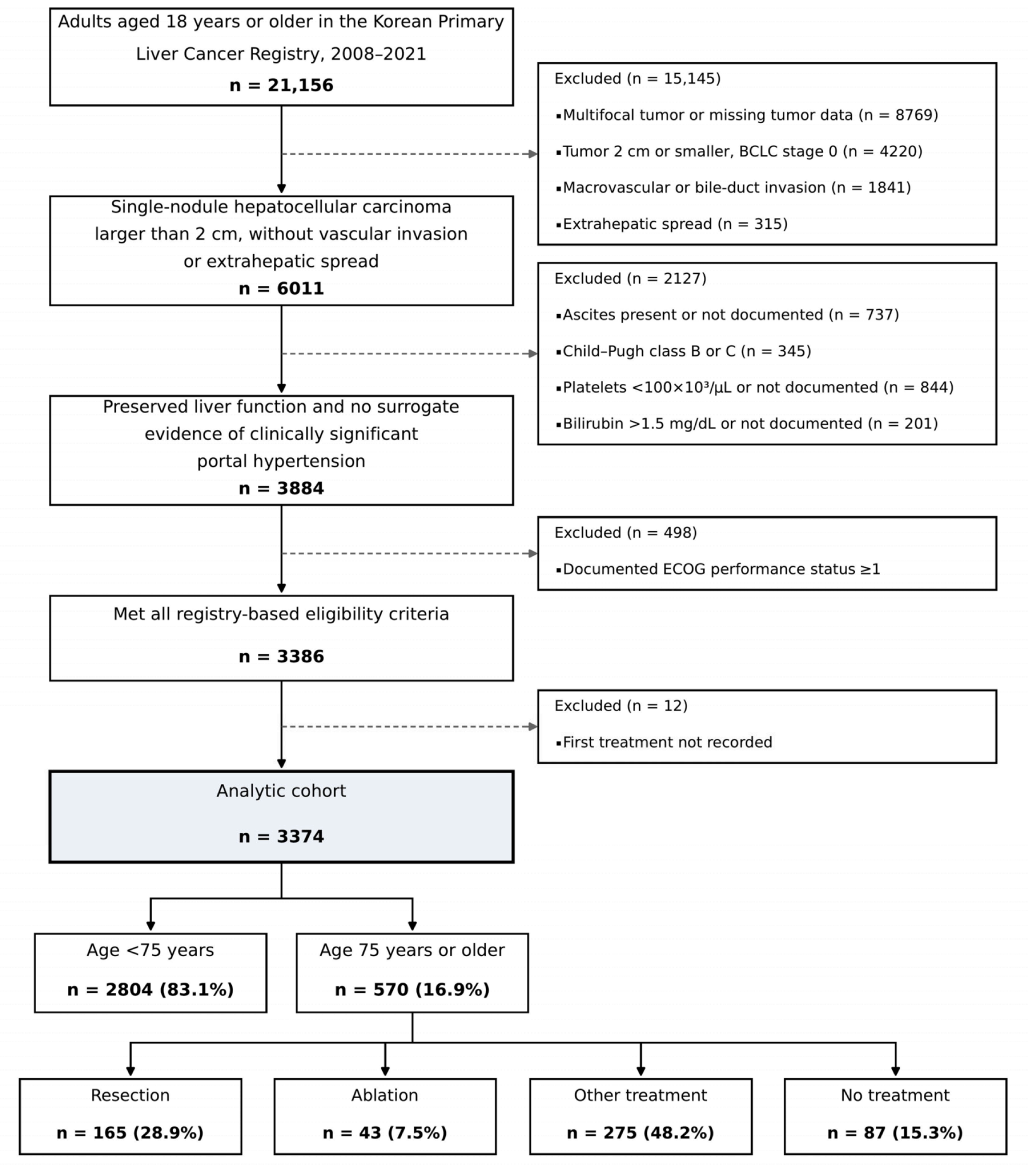
Study flow diagram. Patients were identified in the Korean Primary Liver Cancer Registry between 2008 and 2021. Eligibility followed the criteria of the 2026 BCLC update, applied only to documented values; patients in whom ascites, platelet count, or bilirubin was not recorded were therefore excluded. Twelve patients whose first treatment was not recorded could not be assigned a treatment category. Percentages for the age groups are of the analytic cohort, and percentages for the treatment categories are of patients aged 75 years or older. ECOG, Eastern Cooperative Oncology Group.

We restricted the cohort to single-nodule HCC. Patients with multifocal BCLC-A disease were excluded because the guideline designates liver transplantation as first-line for that subgroup [3]. Of 21,156 adults, 3386 met all criteria; 12 patients in whom the first treatment was not recorded were further excluded because treatment category could not be assigned, leaving 3374 patients (Figure 1).

### 2.3. Treatment Categories

The registry records the first treatment received, and codes the absence of any anti-tumor treatment as a distinct category rather than as a missing value. Patients were therefore classified as having received surgical resection; ablation or liver transplantation; other treatment (transarterial chemoembolization or chemoinfusion, transarterial radioembolization, systemic therapy, or radiotherapy); or no treatment. Because the registry records the modality delivered rather than the intent with which it was given, we name these categories by the treatments they contain rather than as curative or non-curative. No patient aged 75 years or older underwent transplantation, so within the older subgroup this category comprises ablation alone.

### 2.4. Outcome and Time Origin

The outcome was overall survival, measured in every patient from the date of radiological diagnosis, defined as the earlier of the computed tomography and magnetic resonance imaging dates, to death from any cause or to 31 December 2024. A diagnosis date was available for all 3374 patients. Using a single time origin for treated and untreated patients avoids the guarantee-time bias that arises when survival is measured from the date of treatment in treated patients only. Cause of death was obtained by linkage to national death certificates and classified as cancer-related (ICD-10 C22) or other cause.

### 2.5. Covariates and Missing Data

Covariates were sex, hepatitis B and C status, alcohol-related etiology, diabetes, hypertension, body mass index (BMI), platelet count, estimated glomerular filtration rate (eGFR; CKD-EPI 2021 race-free equation), albumin–bilirubin (ALBI) score, maximum tumor diameter, log10-transformed alpha-fetoprotein, and registry era (2008–2012, 2013–2017, 2018–2021) [17]. Age was included as a continuous covariate in all survival and propensity score models; in the logistic model for receipt of resection, age was the exposure of interest and was entered as a binary indicator for age 75 years or older rather than as a continuous covariate. Registry codes for unknown or not tested were treated as missing. Each covariate had less than 10% missing data (Table S1), and the primary models used complete cases (*n* = 2750; 81.5%). Because excluded patients differed from those included, we repeated the analysis after multiple imputation by chained equations (10 imputations, 10 iterations), with the imputation model including the exposure, the event indicator, and the Nelson–Aalen estimate of the cumulative hazard, and pooled estimates by Rubin’s rules [18]. Cirrhosis was recorded only from 2016 onward and was therefore examined in a separate analysis restricted to that period.

### 2.6. Statistical Analysis

We present continuous variables as mean (SD) and categorical variables as *n* (%), compared using *t* test, with the Student or Welch version, selected following a test for equality of variances, and by the chi-square test. We defined older age as 75 years or more, a threshold widely used in recent surgical and oncological cohorts of older patients [19,20] and in HCC [10], and one that approximates the age at which competing non-cancer mortality becomes prominent; consistent with this, age 75 years or older has been associated with shorter overall survival without a corresponding difference in cancer-specific survival [20]. Because the resection rate declined further beyond that threshold, age was additionally modeled as a continuous variable and as 75–79 versus 80 years or older within the older subgroup.

We identified independent predictors of resection by multivariable logistic regression, and estimated overall survival by the Kaplan–Meier method with log-rank comparison and by multivariable Cox regression with Efron handling of ties and Wald confidence intervals. The proportional hazards assumption was assessed with Schoenfeld residuals. Because it was not satisfied for the treatment variable, we report period-specific hazard ratios from a model stratified at 12 and 60 months, a treatment-by-time interaction, and the unadjusted restricted mean survival time (RMST) at 60 months as the principal time-averaged effect measure, which does not require proportional hazards [21]. RMST was estimated from Kaplan–Meier curves within a 60-month window in the full analytic cohort, without covariate adjustment or weighting. For cause-specific outcomes we estimated cumulative incidence functions with Gray’s test and fitted Fine–Gray subdistribution hazard models, treating death from other causes as a competing event [22]. Cumulative incidence functions and Gray’s tests require no covariates and were estimated in the full analytic cohort; the Fine–Gray model was fitted in the complete-case subset.

As a sensitivity analysis we applied inverse probability of treatment weighting (IPTW) based on propensity scores. Propensity scores were estimated by multivariable logistic regression including age, sex, hepatitis B and C status, alcohol-related etiology, diabetes, hypertension, BMI, platelet count, eGFR, ALBI score, maximum tumor diameter, log10-transformed alpha-fetoprotein, and registry era. Stabilized weights were calculated to estimate the average treatment effect and truncated at the 1st and 99th percentiles. Covariate balance before and after weighting was assessed by standardized mean differences (SMDs), with an absolute SMD below 0.1 indicating adequate balance. E-values were calculated to quantify the strength of unmeasured confounding that would be required to explain the observed associations [23], using the approximation for common outcomes. We repeated the primary comparisons in two prespecified sensitivity analyses: restriction to patients with a documented ECOG PS of 0, which tests whether including patients whose performance status was unknown affected the results, and expansion of the cohort to include BCLC stage 0 disease. Cancer-related mortality, which was a separate sensitivity analysis in our earlier work, is now addressed directly by the competing-risk models. All *p* values were two-sided with significance at 0.05. Analyses were performed in R version 4.3.3, and all estimates were independently reproduced from the raw registry export.

## 3. Results

### 3.1. Cohort Characteristics

Of 21,156 adults registered between 2008 and 2021, 3374 met all eligibility criteria (Figure 1). Resection was performed in 1863 patients (55.2%) and 1511 (44.8%) were managed without resection. Within the non-resection group, 250 received ablation (*n* = 238) or liver transplantation (*n* = 12) and 1060 received other treatment, of which transarterial chemoembolization or chemoinfusion accounted for 970 (91.5%); 201 patients received no treatment. All 12 transplant recipients were younger than 75 years (median age 54 years), so no patient in the older subgroup underwent transplantation. Patients undergoing resection were younger, more often hepatitis B-positive, and had marginally better liver function (Table 1). During a median follow-up of 111 months, 1538 patients (45.6%) died.

### 3.2. Use of Resection According to Age

Among 2804 patients younger than 75 years, 1698 (60.6%) underwent resection, compared with 165 of 570 patients 75 years or older (28.9%; *p* < 0.001). Use declined further with age within the older group: 35.2% (122/347) at 75–79 years and 19.3% (43/223) at 80 years or older. Of 405 older patients managed without resection, 87 (21.5%) received no treatment (Table 2).

**Figure 2:**
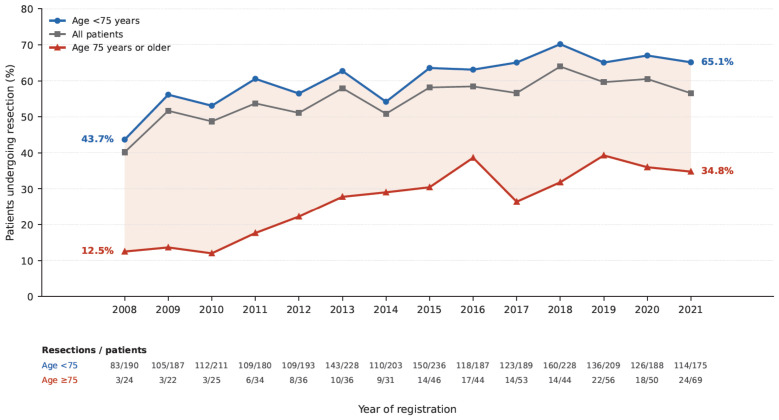
Use of surgical resection over 14 years, according to age group. Symbols show unadjusted annual proportions and the shaded area shows the difference between age groups. The numbers beneath the plot are resections and total patients for each year and age group. Age group by calendar year interaction was not significant (*p* = 0.17).

Use of resection rose in both age strata over the 14-year period (Figure 2): from 43.7% to 65.1% in patients younger than 75 years and from 12.5% to 34.8% in those 75 years or older, with the overall rate rising from 40.2% to 56.6%. The absolute difference between age groups changed little, from 31.2 percentage points in 2008 to 30.4 in 2021, and age group by calendar year interaction was not significant (*p* = 0.17).

### 3.3. Factors Associated with Receipt of Resection

In multivariable logistic regression (*n* = 2750), age 75 years or older was independently associated with lower odds of resection (adjusted odds ratio [aOR] 0.36, 95% CI 0.28–0.46; *p* < 0.001). A higher ALBI score (aOR 0.40, 95% CI 0.32–0.49) and hepatitis C (aOR 0.65, 95% CI 0.48–0.89) were also associated with lower odds, whereas later registry eras were associated with higher odds (2018–2021: aOR 1.63, 95% CI 1.33–2.02). The complete model is provided in Table S2.

### 3.4. Overall Survival

**Figure 3:**
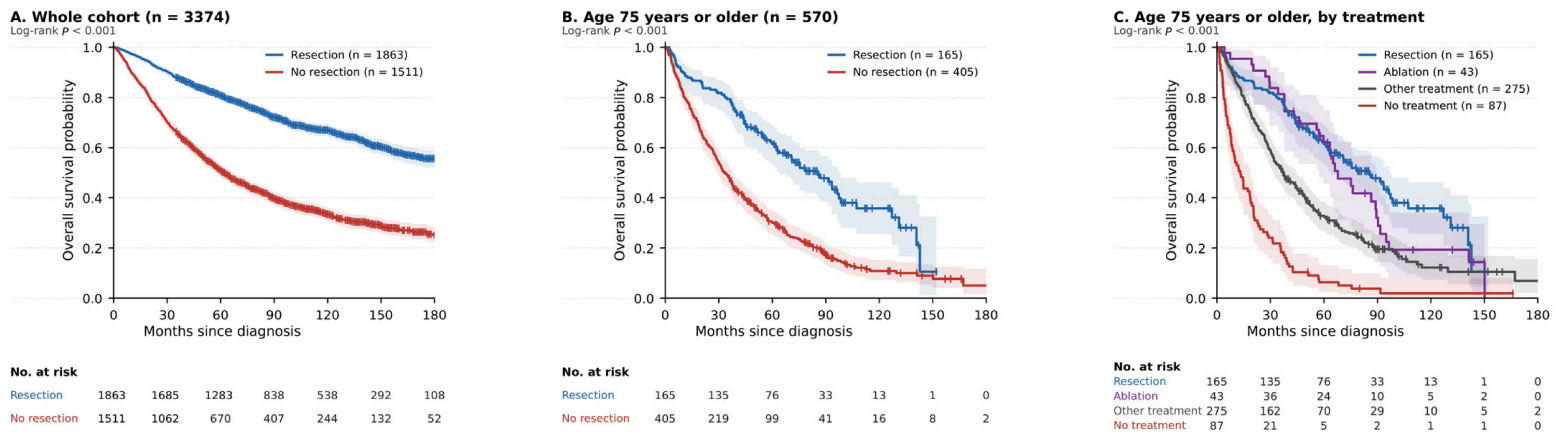
Overall survival according to treatment. (**A**) Whole cohort; (**B**) patients aged 75 years or older; (**C**) patients aged 75 years or older according to treatment category. Survival was measured from the date of radiological diagnosis in all patients. Shaded bands are 95% confidence intervals, vertical tick marks denote censoring, and numbers at risk are shown beneath each panel. Groups were compared by the log-rank test. Ablation, ablation or liver transplantation; other treatment, transarterial chemoembolization or chemoinfusion, transarterial radioembolization, systemic therapy, or radiotherapy.

Five-year overall survival was 80.8% after resection and 50.9% without resection (log-rank *p* < 0.001; Figure 3A). Among patients 75 years or older it was 61.3% and 30.3%, with a median of 85.2 and 33.2 months (Figure 3B). Crude hazard ratios for non-resection versus resection were 2.76 (95% CI 2.49–3.06) overall, 2.43 (2.16–2.74) in patients younger than 75 years, and 2.27 (1.79–2.89) in those 75 years or older. After multivariable adjustment, which included age as a continuous covariate, the association persisted: 2.08 (1.84–2.35) overall, 2.02 (1.76–2.32) below 75 years, and 2.36 (1.77–3.13) at 75 years or older, all *p* < 0.001 (Table 3). Age itself was associated with mortality at 1.04 per year (1.01–1.07, *p* = 0.02).

The proportional hazards assumption was not satisfied for the treatment variable (*p* = 0.03), and a treatment-by-time interaction was significant (*p* = 0.04). Period-specific estimates showed a consistent direction with attenuation over time: in the whole cohort the adjusted hazard ratio was 2.43 (1.73–3.40) within 12 months, 2.30 (1.95–2.71) between 12 and 60 months, and 1.69 (1.38–2.07) thereafter. Among older patients the corresponding estimates were 1.48 (0.83–2.63), 3.12 (2.12–4.58), and 1.78 (1.02–3.10). The estimate for the first period was imprecise and its confidence interval included the null; early perioperative risk offsetting the benefit of resection during the first year is one possible explanation, although other explanations cannot be excluded. Expressed as unadjusted restricted mean survival time at 60 months, mean survival within the first 5 years was 10.6 months longer after resection in the whole cohort (95% CI 9.4–11.8; 54.2 versus 43.6 months) and 13.3 months longer among patients 75 years or older (9.7–16.9; 48.0 versus 34.7 months).

### 3.5. Treatment Categories Among Older Patients

Survival among older patients differed markedly across treatment categories (log-rank *p* < 0.001; Figure 3C; Table S4). Median overall survival was 85.2 months after resection (5-year survival 61.3%), 67.9 months after ablation (62.1%), 37.5 months after other treatment (32.9%), and 13.1 months with no treatment (6.5%). With resection as the reference, adjusted hazard ratios were 1.97 (95% CI 1.21–3.21; *p* = 0.007) for ablation, which is consistent with meta-analytic comparisons of ablation and resection in older patients with HCC [24], 2.16 (1.61–2.90; E-value 2.79) for other treatment, and 4.97 (3.26–7.57; *p* < 0.001; E-value 5.34) for no treatment. Explaining away the last of these associations would require an unmeasured confounder associated with both treatment and mortality by a risk ratio of about 5.3, which we consider unlikely for any single measured domain, although such a confounder cannot be excluded.

### 3.6. Cause-Specific Mortality and Competing Risks

Among 1538 deaths, 1169 were cancer-related and 369 were attributed to other causes; a cause was recorded for every death. Five-year cumulative incidence of cancer-related death was 15.6% after resection and 39.6% without resection, and that of other-cause death 3.6% and 9.5% (Gray’s test *p* < 0.001 for both; Figure S1). Among patients 75 years or older, cumulative incidence of cancer-related death was 30.1% and 54.4% (*p* < 0.001), whereas other-cause death did not differ between treatment groups (8.6% and 15.3%; *p* = 0.78). In a Fine–Gray model fitted in the complete-case subset (*n* = 2750) and accounting for other-cause death as a competing event, non-resection remained associated with cancer-related death (subdistribution hazard ratio 2.14, 95% CI 1.85–2.47; E-value 2.77).

### 3.7. Sensitivity Analyses

After IPTW, all covariates including age were balanced; the largest imbalance, which was for age, fell from a standardized mean difference of 0.510 before weighting to 0.008 after, and no covariate exceeded 0.012 (Table S5), and the weighted hazard ratio for non-resection versus resection was 1.86 (95% CI 1.64–2.11). Patients excluded from the complete-case analysis were older and more often managed without resection (Table S6), but multiple imputation gave nearly identical estimates (2.08, 95% CI 1.87–2.32 overall; 2.13, 1.65–2.75 at 75 years or older). In the 1492 patients registered from 2016 onward, in whom cirrhosis was documented in 99.4%, the adjusted hazard ratio for non-resection was 1.90 (1.52–2.36) before and 1.84 (1.47–2.29) after adding cirrhosis to the model, with cirrhosis itself associated with mortality (1.33, 1.06–1.67; *p* = 0.01). The two prespecified sensitivity analyses were also consistent (Table S7, Figure S2). Restricting the cohort to the 2390 patients with a documented ECOG PS of 0 gave an adjusted odds ratio for resection of 0.40 (95% CI 0.29–0.53) and an adjusted hazard ratio for no resection of 2.27 (1.62–3.19) among older patients, with 5-year survival of 62.7% after resection and 10.6% with no treatment. Expanding the cohort to 5256 patients by including BCLC stage 0 gave an adjusted odds ratio of 0.38 (0.30–0.47) and an adjusted hazard ratio of 2.17 (1.66–2.84), with 5-year survival of 62.5% and 10.4%. In both analyses the adjusted hazard ratio for no treatment versus resection remained close to 5 (4.98 and 4.39).

## 4. Discussion

In this 14-year nationwide cohort of 3374 patients who met every registry-based eligibility criterion for resection, three observations stand out. Resection was used in fewer than one in three patients aged 75 years or older, compared with three in five younger patients, and this absolute difference persisted throughout the study period. More than one in five older patients managed without resection received no treatment at all. Finally, adjusted mortality risk rose as treatment intensity decreased, and explaining away the difference between resection and no treatment would require an unmeasured confounder associated with both treatment and mortality by a risk ratio of about 5.3 (E-value 5.34). We consider a single unmeasured factor of that magnitude unlikely, although it cannot be excluded.

Most previous studies of age-related differences in HCC treatment have drawn on mixed populations spanning all stages and degrees of liver dysfunction, in which the influence of age cannot be separated from that of tumor burden or hepatic reserve. By restricting the analysis to patients who met identical eligibility criteria, we reduced this source of confounding, although unmeasured contraindications could not be excluded. The pattern we observed is consistent with longstanding evidence that curative therapy is underused in HCC, including a recent US cohort in which only 27% to 43% of patients received curative treatment [25], and with European data showing that older patients are both treated less aggressively and carry a worse adjusted long-term prognosis, most apparent in early-stage disease [26]. An earlier analysis of the same registry reported that patients 65 years or older were less likely to be treated than younger patients [27], but that work spanned all stages and did not isolate patients eligible for resection; to our knowledge, survival across these treatment categories has not previously been compared in an older population restricted to patients meeting uniform eligibility criteria for resection.

That age alone need not preclude treatment is supported across modalities. A meta-analysis of resection in older patients with HCC, in whom elderly was defined as 65 years or older, found that among studies reporting multivariable-adjusted estimates, overall survival did not differ significantly between older and younger patients (hazard ratio 1.26, 95% CI 0.92–1.74) [28]. The same pattern holds for systemic therapy: in a Japanese real-world cohort treated with atezolizumab plus bevacizumab, survival did not differ between patients 75 years or older and younger patients [29], and a French cohort found comparable survival with poor outcomes confined to those with severely impaired liver function [9]. Against this evidence, the persistence of an age-related difference in the use of resection, even after adjustment for liver function, renal function, comorbidity, and tumor burden, suggests that age influences treatment allocation independently of expected benefit. Consistent with this, although resection in older patients rose from 12.5% to 34.8% over the study period, the difference from younger patients remained near 30 percentage points throughout. Treatment patterns for hepatocellular carcinoma in Korea shifted substantially over the same years [30], yet that shift did not translate into a narrowing of the age-related difference in the use of resection.

This analysis has several strengths. Vital status and cause of death were complete through the 2024 cutoff via linkage to national death records, eligibility criteria were prespecified, and survival was measured from a single time origin in all patients. Because the proportional hazards assumption was not satisfied, we report period-specific hazard ratios and restricted mean survival time, which quantify the association in months of life rather than as a single ratio, and we accounted for competing non-cancer mortality, which is particularly relevant in an older population. Adjustment was broad, incorporating renal function, body mass index, comorbidity, and liver function in place of single collinear laboratory values, capturing several physiological domains relevant to fitness for surgery [16,31], and the findings were consistent across IPTW, multiple imputation, and cirrhosis-adjusted analyses.

Several limitations should be acknowledged. First, this is an observational study, and the registry does not record why a given patient did not undergo resection; unmeasured factors such as frailty, cognitive status, patient preference, and the surgical team’s judgment may have contributed. The same factors bear on the survival comparison: patients with subclinical deterioration or limited life expectancy may have been steered away from treatment, so that the absence of active treatment partly marks a worse underlying prognosis rather than fully causing the excess mortality. Adjustment, weighting, and E-values address measured confounding factors but cannot remove this selection, and the estimates should be read as associations rather than as treatment effects. Second, eligibility was established from registry variables. CSPH was inferred from absent ascites, Child–Pugh class A, and platelet count rather than measured directly, and this surrogate could not be validated against hepatic venous pressure gradient or endoscopic findings within the registry; some patients classified as eligible may therefore have had portal hypertension, and others may have had contraindications, such as cardiopulmonary disease or anesthetic risk, that the registry does not capture. For the same reason, we describe the cohort as meeting registry-based eligibility criteria rather than as being unequivocally suitable for surgery. Third, the registry records neither tumor location, nor the extent of the resection required, nor the anticipated future liver remnant, and these anatomical and technical factors weigh heavily in the decision to operate. Compression of the hepatic or portal vein by the tumor is associated with microvascular invasion and satellite nodules [32], and when a hemihepatectomy is required, resection of the middle hepatic vein lengthens the operation and increases blood loss and postoperative transaminase levels, although it does not appear to affect long-term survival [33]; a peripheral tumor amenable to a limited resection and a deep-seated tumor requiring a major hepatectomy are therefore very different propositions in an older patient with limited functional reserve, even though both satisfy the same guideline criteria. Fourth, cirrhosis was recorded only from 2016 onward, although adding it to the model in that period did not materially change the estimate. Fifth, performance status was incompletely recorded, and ECOG PS is, in any case, too coarse to capture frailty; the 2026 guideline now recommends systematic geriatric assessment in older candidates [3]. Finally, treatment was abstracted from records at the diagnosing hospital, so treatment delivered elsewhere may not have been captured; such misclassification would move treated patients into the untreated group and would attenuate rather than create the difference we report.

## 5. Conclusions

Among patients meeting registry-based eligibility criteria for resection, those aged 75 years or older underwent resection far less often than younger patients, and one in five of those managed without resection received no treatment at all. Adjusted mortality risk rose as treatment intensity decreased, and mean survival within the first 5 years was 13.3 months longer in patients who underwent resection. Because the difference in resection use persisted after adjustment for age, comorbidity, liver function, and tumor burden, meeting eligibility criteria warrants consideration of resection, and referral for active treatment when resection is judged unsuitable. Recording why resection was not offered would help distinguish appropriate selection from lower use in future registry analyses.

## Figures and Tables

**Table 1:** Baseline characteristics of patients meeting registry-based eligibility criteria for resection, according to initial treatment.

Variable	Total (*n* = 3374)	Surgical Resection (*n* = 1863)	No Resection (*n* = 1511)	*p* Value
Age, years	62.6 ± 11.7	59.9 ± 11.1	65.9 ± 11.5	<0.001
<65	1860 (55.1%)	1194 (64.1%)	666 (44.1%)	
65–74	944 (28.0%)	504 (27.1%)	440 (29.1%)	
75–79	347 (10.3%)	122 (6.5%)	225 (14.9%)	
≥80	223 (6.6%)	43 (2.3%)	180 (11.9%)	
Female sex	684 (20.3%)	356 (19.1%)	328 (21.7%)	0.068
Body mass index, kg/m^2^	24.4 ± 3.3	24.4 ± 3.2	24.2 ± 3.5	0.076
Hepatitis B virus	1938 (59.2%)	1206 (65.6%)	732 (51.0%)	<0.001
Hepatitis C virus	277 (9.0%)	105 (6.0%)	172 (12.8%)	<0.001
Alcohol-related etiology	1016 (30.7%)	530 (28.9%)	486 (32.9%)	0.015
Diabetes mellitus	981 (29.2%)	499 (26.8%)	482 (32.1%)	<0.001
Hypertension	1547 (46.1%)	809 (43.5%)	738 (49.3%)	0.001
Maximum tumor diameter, cm	4.7 ± 2.9	4.5 ± 2.6	4.9 ± 3.2	<0.001
Platelet count, ×10^3^/μL	189.1 ± 67.0	191.8 ± 63.9	185.8 ± 70.5	0.011
eGFR, mL/min/1.73 m^2^	88.2 ± 19.7	90.9 ± 18.1	84.8 ± 21.0	<0.001
ALBI score	−2.8 ± 0.4	−2.9 ± 0.4	−2.7 ± 0.4	<0.001
log10 alpha-fetoprotein, ng/mL	1.4 ± 1.2	1.4 ± 1.1	1.4 ± 1.2	0.670

Continuous variables are presented as mean ± SD and categorical variables as *n* (%). *p* values were obtained by the *t* test for continuous variables and the chi-square test for categorical variables. ALBI, albumin–bilirubin; eGFR, estimated glomerular filtration rate.

**Table 2:** Baseline characteristics of patients aged 75 years or older, according to initial treatment.

Variable	Total (*n* = 570)	Surgical Resection (*n* = 165)	No Resection (*n* = 405)	*p* Value
Age, years	79.3 ± 3.7	78.1 ± 3.1	79.7 ± 3.9	<0.001
75–79	347 (60.9%)	122 (73.9%)	225 (55.6%)	
≥80	223 (39.1%)	43 (26.1%)	180 (44.4%)	
Female sex	153 (26.8%)	42 (25.5%)	111 (27.4%)	0.709
Body mass index, kg/m^2^	23.9 ± 3.5	24.3 ± 3.3	23.8 ± 3.6	0.090
Hepatitis B virus	118 (21.9%)	39 (24.2%)	79 (20.9%)	0.459
Hepatitis C virus	80 (15.6%)	21 (14.0%)	59 (16.3%)	0.604
Alcohol-related etiology	152 (27.4%)	44 (27.3%)	108 (27.5%)	1.000
Diabetes mellitus	232 (41.1%)	62 (37.6%)	170 (42.5%)	0.323
Hypertension	365 (64.7%)	107 (64.8%)	258 (64.7%)	1.000
Maximum tumor diameter, cm	5.4 ± 3.1	5.1 ± 2.7	5.5 ± 3.2	0.190
Platelet count, ×10^3^/μL	199.8 ± 74.7	197.5 ± 57.0	200.8 ± 80.9	0.588
eGFR, mL/min/1.73 m^2^	73.5 ± 18.4	75.7 ± 17.3	72.5 ± 18.8	0.051
ALBI score	−2.7 ± 0.4	−2.8 ± 0.4	−2.7 ± 0.4	<0.001
log10 alpha-fetoprotein, ng/mL	1.3 ± 1.2	1.3 ± 1.1	1.4 ± 1.2	0.484

Abbreviations as in Table 1.

**Table 3:** Overall survival according to treatment, in the whole cohort and by age group.

Group	No. of Patients	Median OS, Months	5-Year OS, %	Crude HR (95% CI)	Adjusted HR (95% CI)	IPTW HR (95% CI)
Whole cohort (*n* = 3374)						
Surgical resection	1863	Not reached	80.8	Reference	Reference	Reference
No resection	1511	61.8	50.9	2.76 (2.49–3.06)	2.08 (1.84–2.35)	1.86 (1.64–2.11)
Age < 75 years (*n* = 2804)						
Surgical resection	1698	Not reached	82.6	Reference	Reference	Reference
No resection	1106	82.9	58.3	2.43 (2.16–2.74)	2.02 (1.76–2.32)	
Age ≥ 75 years (*n* = 570)						
Surgical resection	165	85.2	61.3	Reference	Reference	Reference
No resection	405	33.2	30.3	2.27 (1.79–2.89)	2.36 (1.77–3.13)	1.84 (1.42–2.38)
Treatment category, age ≥ 75 years						
Surgical resection	165	85.2	61.3		Reference	
Ablation	43	67.9	62.1		1.97 (1.21–3.21)	
Other treatment	275	37.5	32.9		2.16 (1.61–2.90)	
No treatment	87	13.1	6.5		4.97 (3.26–7.57)	

Survival was measured from the date of radiological diagnosis in all patients. Adjusted models included age, sex, hepatitis B and C status, alcohol-related etiology, diabetes, hypertension, body mass index, platelet count, eGFR, ALBI score, maximum tumor diameter, log10-transformed alpha-fetoprotein, and registry era, with Efron handling of ties and Wald confidence intervals. Inverse probability of treatment weighting used stabilized weights for the average treatment effect, truncated at the 1st and 99th percentiles. Because the proportional hazards assumption was not satisfied for the treatment variable, hazard ratios represent time-averaged associations and should be read together with the period-specific estimates in Table S3 and the restricted mean survival time reported in the text. CI, confidence interval; HR, hazard ratio; IPTW, inverse probability of treatment weighting; OS, overall survival.

## Data Availability

The data analyzed in this study were provided by the Korean Primary Liver Cancer Registry under a data-use agreement and are not publicly available; the authors are not permitted to redistribute them. Access is granted by the Korean Liver Cancer Association to researchers whose applications are approved. The R code used for the analyses is available from the corresponding author on request.

## Supplementary Materials

The following supporting information can be downloaded at: https://www.mdpi.com/article/10.3390/cancers18152457/s1, Table S1: Missing data by variable in the analytic cohort; Table S2: Complete multivariable logistic regression model for receipt of surgical resection; Table S3: Period-specific adjusted hazard ratios for no resection versus surgical resection; Table S4: Characteristics of patients aged 75 years or older according to treatment category; Table S5: Covariate balance before and after inverse probability of treatment weighting; Table S6: Comparison of patients included in and excluded from the complete-case analysis; Table S7: Prespecified sensitivity analyses; Figure S1: cumulative incidence of cancer-related and other-cause death according to treatment, in the whole cohort (A) and in patients aged 75 years or older (B); Figure S2: overall survival by treatment category among patients aged 75 years or older, across the prespecified sensitivity analyses.

## Abbreviations

aHR, adjusted hazard ratio; ALBI, albumin-bilirubin; aOR, adjusted odds ratio; BCLC, Barcelona Clinic Liver Cancer; BMI, body mass index; CI, confidence interval; CIF, cumulative incidence function; CKD-EPI, Chronic Kidney Disease Epidemiology Collaboration; CSPH, clinically significant portal hypertension; ECOG PS, Eastern Cooperative Oncology Group performance status; eGFR, estimated glomerular filtration rate; HCC, hepatocellular carcinoma; IPTW, inverse probability of treatment weighting; KCCR, Korea Central Cancer Registry; KLCA, Korean Liver Cancer Association; KPLCR, Korean Primary Liver Cancer Registry; RECORD, REporting of studies Conducted using Observational Routinely collected health Data; RMST, restricted mean survival time; SMD, standardized mean difference; STROBE, Strengthening the Reporting of Observational Studies in Epidemiology.
