# Supplementary material for: Discrepancy Between Eligibility and Practice: A 14-Year Nationwide Study on Curative Resection in Elderly Hepatocellular Carcinoma Patients
Source: Cancers (Basel). 2026 Jul 30;18(15):2457. doi: 10.3390/cancers18152457 (PMC13465431; doi:10.3390/cancers18152457)
Supplement: Supplementary file 1 [file cancers-18-02457-s001.zip › cancers-4460278-supplementary.pdf]

Supplementary Materials

Table S1. Missing data by variable in the analytic cohort.

Variable	No. missing	Missing, %
Age	0	0.0%
Sex	0	0.0%
Hepatitis B virus	100	3.0%
Hepatitis C virus	285	8.4%
Alcohol-related etiology	63	1.9%
Diabetes mellitus	14	0.4%
Hypertension	18	0.5%
Body mass index	156	4.6%
Platelet count	0	0.0%
eGFR	14	0.4%
ALBI score	6	0.2%
Maximum tumor diameter	0	0.0%
Alpha-fetoprotein	171	5.1%
Registry era	0	0.0%
Cirrhosis	1891	56.0%

Cirrhosis was recorded in the registry only from 2016 onward and was therefore analyzed separately in patients registered during that period.

Table S2. Complete multivariable logistic regression model for receipt of surgical resection.

Variable	Adjusted odds ratio (95% CI)	P value
Age ≥75 years	0.36 (0.28–0.46)	<0.001
Female sex	0.90 (0.73–1.12)	0.359
Hepatitis B virus	1.26 (1.04–1.52)	0.020
Hepatitis C virus	0.65 (0.48–0.89)	0.007
Alcohol-related etiology	0.80 (0.66–0.96)	0.018
Diabetes mellitus	0.89 (0.74–1.08)	0.252
Hypertension	0.91 (0.76–1.10)	0.340
Body mass index, per 1 kg/m²	1.02 (1.00–1.05)	0.109
Platelet count, per 1×10³/μL	1.00 (1.00–1.00)	0.001
eGFR, per 1 mL/min/1.73 m²	1.00 (1.00–1.01)	0.039
ALBI score, per 1 unit	0.40 (0.32–0.49)	<0.001
Maximum tumor diameter, per 1 cm	0.97 (0.94–1.00)	0.082
log10 alpha-fetoprotein, per 1 unit	1.03 (0.95–1.11)	0.471
Registry era 2013–2017	1.39 (1.15–1.69)	<0.001
Registry era 2018–2021	1.63 (1.33–2.02)	<0.001

n = 2,750 patients with complete covariate data. ALBI, albumin-bilirubin; CI, confidence interval; eGFR, estimated glomerular filtration rate.

Table S3. Period-specific adjusted hazard ratios for no resection versus surgical resection.

Cohort	Period	Adjusted HR (95% CI)
Whole cohort	0–12 months	2.43 (1.73–3.40)
Whole cohort	12–60 months	2.30 (1.95–2.71)
Whole cohort	>60 months	1.69 (1.38–2.07)
Age ≥75 years	0–12 months	1.48 (0.83–2.63)
Age ≥75 years	12–60 months	3.12 (2.12–4.58)
Age ≥75 years	>60 months	1.78 (1.02–3.10)

Estimates were obtained from a Cox model stratified by follow-up period with a treatment-by-period interaction, adjusted for the covariates listed in Table 3. A treatment-by-time interaction using the logarithm of follow-up time was significant (P = 0.04).

Table S4. Characteristics of patients aged 75 years or older according to treatment category.

Variable	Total (n=570)	Resection (n=165)	Ablation (n=43)	Other treatment (n=275)	No treatment (n=87)	P value
Age, years	79.3±3.7	78.1±3.1	79.0±3.9	79.4±3.7	81.0±4.2	<0.001
75–79	347 (60.9%)	122 (73.9%)	30 (69.8%)	161 (58.5%)	34 (39.1%)	
≥80	223 (39.1%)	43 (26.1%)	13 (30.2%)	114 (41.5%)	53 (60.9%)	
Female sex	153 (26.8%)	42 (25.5%)	16 (37.2%)	68 (24.7%)	27 (31.0%)	0.270
Body mass index, kg/m²	23.9±3.5	24.3±3.3	23.9±3.6	24.1±3.6	22.5±3.5	0.005
Hepatitis B virus	118 (21.9%)	39 (24.2%)	9 (22.0%)	53 (20.7%)	17 (21.0%)	0.858
Hepatitis C virus	80 (15.6%)	21 (14.0%)	9 (24.3%)	37 (15.1%)	13 (16.2%)	0.475
Alcohol-related etiology	152 (27.4%)	44 (27.3%)	11 (26.8%)	74 (27.3%)	23 (28.4%)	0.997
Diabetes mellitus	232 (41.1%)	62 (37.6%)	14 (32.6%)	124 (45.3%)	32 (38.6%)	0.229
Hypertension	365 (64.7%)	107 (64.8%)	28 (65.1%)	183 (66.8%)	47 (57.3%)	0.478
Maximum tumor diameter, cm	5.4±3.1	5.1±2.7	3.0±0.6	5.5±3.2	6.6±3.4	<0.001
Platelet count, ×10³/μL	199.8±74.7	197.5±57.0	167.3±51.5	196.0±74.5	232.5±100.7	<0.001
eGFR, mL/min/1.73 m²	73.5±18.4	75.7±17.3	72.6±15.9	72.4±18.7	72.9±20.6	0.307
ALBI score	-2.7±0.4	-2.8±0.4	-2.8±0.3	-2.7±0.4	-2.5±0.4	<0.001
log10 alpha-fetoprotein, ng/mL	1.3±1.2	1.3±1.1	0.8±0.7	1.4±1.3	1.5±1.2	0.023
Died during follow-up	414 (72.6%)	84 (50.9%)	32 (74.4%)	214 (77.8%)	84 (96.6%)	<0.001

Continuous variables are presented as mean±SD and categorical variables as n (%). P values were obtained by one-way analysis of variance for continuous variables and by the chi-square test for categorical variables. No patient in this age group underwent liver transplantation, so the ablation category comprises local ablation only. Other treatment comprises transarterial chemoembolization or chemoinfusion, transarterial radioembolization, systemic therapy, and radiotherapy. ALBI, albumin-bilirubin; eGFR, estimated glomerular filtration rate.

Table S5. Covariate balance before and after inverse probability of treatment weighting.

Covariate	SMD before weighting	SMD after weighting
Age	0.510	0.008
Female sex	0.049	-0.008
Hepatitis B virus	-0.318	-0.009
Hepatitis C virus	0.207	0.007
Alcohol-related etiology	0.082	0.010
Diabetes mellitus	0.117	0.007
Hypertension	0.137	-0.005
Body mass index	-0.070	0.005
Platelet count	-0.092	-0.004
eGFR	-0.265	-0.007
ALBI score	0.453	0.012
Maximum tumor diameter	0.126	-0.006
log10 alpha-fetoprotein	-0.012	-0.004
Registry era 2013–2017	-0.038	-0.006
Registry era 2018–2021	-0.137	-0.004

Standardized mean differences (SMDs) compare patients managed without resection with those undergoing resection. The propensity score model included every covariate listed, including age. Stabilised weights for the average treatment effect were truncated at the 1st and 99th percentiles (range 0.52 to 2.65; mean 1.00). An absolute SMD below 0.1 indicates adequate balance.

Table S6. Comparison of patients included in and excluded from the complete-case analysis.

Variable	Included (n=2750)	Excluded (n=624)	P value
Age, years	62.3	63.9	0.002
Age ≥75 years	15.7%	22.1%	<0.001
Female sex	20.1%	20.8%	0.741
No resection	41.3%	60.1%	<0.001
Died	43.4%	55.3%	<0.001
Maximum tumor diameter, cm	4.7	4.7	0.850
ALBI score	-2.9	-2.8	<0.001
Platelet count	189.6	187.0	0.404
Follow-up, months	81.1	71.1	<0.001

Patients were excluded when any covariate required for the adjusted models was missing. Continuous variables are shown as means.

Table S7. Prespecified sensitivity analyses.

Analysis	No. of patients	Resection, all	Resection, age ≥75 y	aOR for age ≥75 y	Adjusted HR, no resection (all)	Adjusted HR, no resection (age ≥75 y)	5-y survival, resection (age ≥75 y)	5-y survival, no treatment (age ≥75 y)	Adjusted HR, no treatment vs resection (age ≥75 y)
Main analysis	3,374	55.2%	28.9%	0.36 (0.28–0.46)	2.08 (1.84–2.35)	2.36 (1.77–3.13)	61.3%	6.5%	4.97 (3.26–7.57)
ECOG performance status 0 only	2,390	59.5%	34.6%	0.40 (0.29–0.53)	2.06 (1.79–2.38)	2.27 (1.62–3.19)	62.7%	10.6%	4.98 (2.88–8.62)
BCLC stage 0 and A included	5,256	47.2%	25.1%	0.38 (0.30–0.47)	2.01 (1.81–2.24)	2.17 (1.66–2.84)	62.5%	10.4%	4.39 (2.98–6.48)

Each analysis repeats the primary comparisons in a differently defined cohort. Adjusted models included the covariates listed in Table 3. aOR, adjusted odds ratio; HR, hazard ratio.

Figure S1. Cumulative incidence of cancer-related and other-cause death according to treatment, in the whole analytic cohort of 3,374 patients (A) and in the 570 patients aged 75 years or older (B).

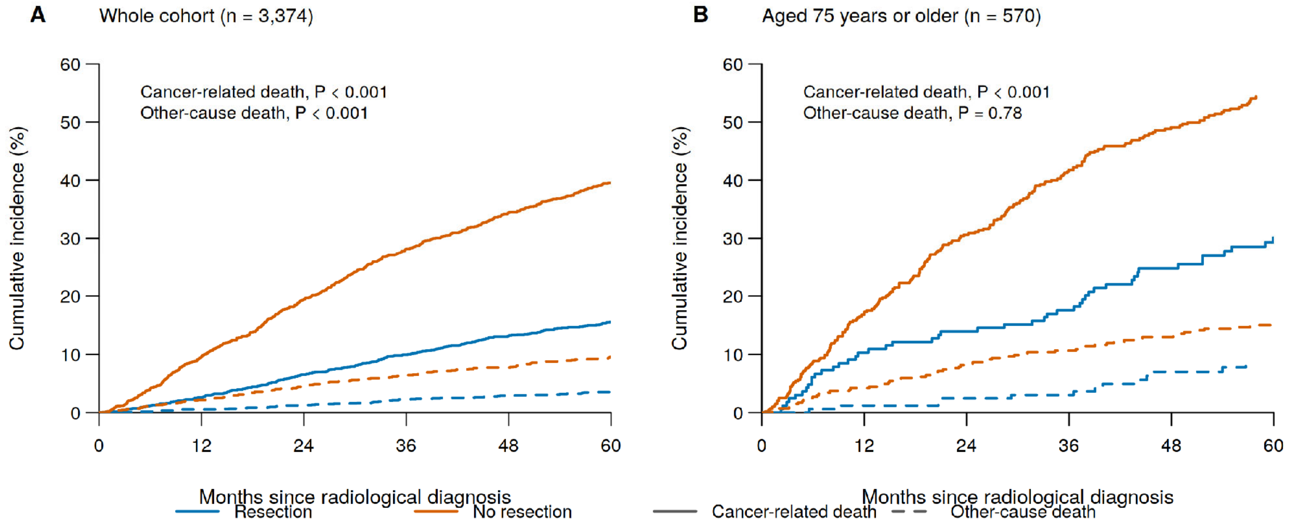

Cumulative incidence functions were estimated with death from the other cause treated as a competing event. Solid lines denote cancer-related death (ICD-10 C22) and dashed lines other-cause death. Survival time was measured from the date of radiological diagnosis. Groups were compared by Gray's test.

Figure S2. Overall survival by treatment category among patients aged 75 years or older, across the prespecified sensitivity analyses.

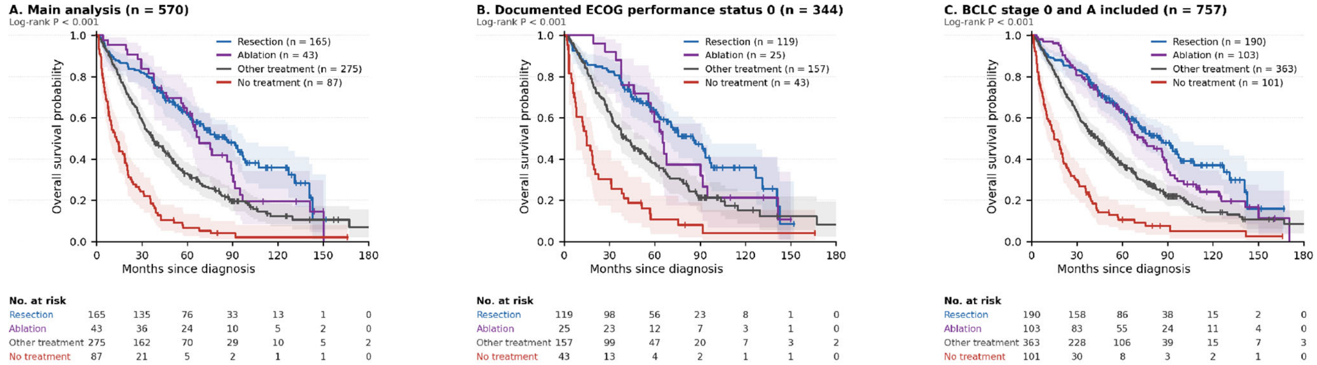

Panel A, main analysis; panel B, restricted to patients with a documented Eastern Cooperative Oncology Group performance status of 0; panel C, cohort expanded to include BCLC stage 0 (tumors 2 cm or smaller). Survival was measured from the date of radiological diagnosis. Shaded bands are 95% confidence intervals, vertical tick marks denote censoring, and numbers at risk are shown beneath each panel. Groups were compared by the log-rank test. Ablation, ablation or liver transplantation; other treatment, transarterial chemoembolization or chemoinfusion, transarterial radioembolization, systemic therapy, or radiotherapy.
